# Sensor Acquisition and Allocation for Real-Time Monitoring of Articulated Construction Equipment in Digital Twins

**DOI:** 10.3390/s22197635

**Published:** 2022-10-09

**Authors:** Sanat A. Talmaki, Vineet R. Kamat

**Affiliations:** Department of Civil and Environmental Engineering, University of Michigan, Ann Arbor, MI 48109-2125, USA

**Keywords:** digital twin, monitoring, visualization, earthmoving, excavation safety

## Abstract

The visibility available to an equipment operator on a dynamic construction site can often be blocked by various obstacles such as materials, temporary or permanent facilities, other equipment, and workers. Equipment monitoring in real-time digital twins can thus play a crucial role in accident prevention. This paper develops a scalable technical approach and presents a prototype application framework for transmitting real world sensor data to update 3D equipment models inside a graphical digital twin for concurrent visualization of a monitored construction operation. The developed framework and workflow can be extended to visualize any construction operation, as it occurs, inside a dynamic 3D world simply by outfitting the real equipment with appropriate sensors and connecting them to their virtual counterparts. The implemented proof-of-concept interface is described in the context of a real-time 3D digital twin for assisting excavator operators prevent unintended strikes with underground utilities. Experiments to validate the proposed technical approach by simulating the real-time motion of a backhoe loader’s articulated arm using orientation sensors installed on its boom, stick, and bucket are described. The experimental results characterize the scope and potential reasons for spatio-temporal discrepancies that can occur between a monitored real operation and its replicated digital twin. The effect of an operator warning mechanism based on preset safety thresholds is also investigated and described.

## 1. Introduction

In fields such as mining, quarrying, and construction, equipment monitoring can play a crucial role in accident prevention [1]. Construction jobsites, in particular, are occupied by workers and equipment, often belonging to different sub-contractors [2]. The visibility available to an operator on a dynamic construction site can often be blocked by various obstacles such as materials, temporary or permanent facilities, other equipment, and even workers [3]. It has been documented that vision is the primary source of information for equipment operators to avoid collisions with other entities on construction jobsites [4]. The importance of clear, unobstructed vision, coupled with the inherent poor visibility that operators of equipment such as dump trucks, loaders, and excavators deal with due to blind spots [5] and other issues, suggests that equipment monitoring and active visual guidance can play a critical role in jobsite safety [6].

Operators in some engineering tasks have the added cognitive burden of using judgment and estimation while performing their activities. Examples of this are excavation in the presence of buried utilities, drilling in reinforced concrete slabs, and tunnel boring operations that involve manipulation around occluded infrastructure, such as underground utilities and ductwork embedded in concrete walls. In the case of buried utilities, any errors on the part of an operator can rupture a gas line leading to an explosion, damage a water or sewer main leading to flooding, or sever an electric conduit resulting in the risk of electrocution or loss of power to homes and businesses [7,8,9,10].

The type of equipment that can be monitored can vary from jobsite to jobsite, and even a single jobsite can have monitoring requirements that span different categories of equipment. The type of sensing mechanism used to record the position and orientation of equipment is also an operation- and equipment-dependent parameter. Thus, any monitoring framework intended for jobsite safety via active visual guidance must be scalable and generic in order to be capable of monitoring equipment and operations across a broad range of conditions and engineering activities.

Equipment monitoring on a construction jobsite can be achieved using various approaches. Video surveillance, additional supervision, computer vision, Global Positioning System (GPS), and Radio Frequency Identification (RFID) are all examples of monitoring and control methods [11,12]. This paper describes the limitations of these existing methods, and introduces a sensor-based, real-time 3D visualization and geometric proximity monitoring method. Through this method, an equipment operator is provided with a combination of visual assistance through real-time 3D visualization in a digital twin, and audio-visual warnings through proximity monitoring, that together can help prevent any impending interference, collision, or accident. The following sections describe the details of the proposed computational framework for providing real-time sensor updates by mapping field-based sensor data to corresponding equipment components in a 3D digital twin.

One construction operation in which equipment operators are routinely challenged on their skill and judgment, and often in equal measure, is excavation, especially when carried out in the presence of underground utilities. Excavation is thus selected as an illustrative proof-of-concept example of an operation where monitoring of equipment can provide critical assistance to operators, to help them perform the activity safely. As the existing buried utilities are covered by earth and soil, an operator cannot be certain of their location in relation to the equipment’s end-effector (a bucket in the case of a backhoe or excavator). Excavation has thus become one of the leading causes of damage to underground utilities [13,14].

Whenever a buried utility is struck during excavation, it can result in loss of life, injuries, damage to property, and a disruption to general life and commerce. Based on statistics compiled during the Census of Fatal Occupational Injuries, published by the U.S. Bureau of Labor Statistics, there were 3645 contact-driven fatalities that occurred during construction in the time period between 2009 and 2018 [15]. Due to its inherent operational dangers, excavation is thus an ideal construction process to highlight the benefits of equipment monitoring for improving safety.

In the presented research, the authors’ instrument is a backhoe loader with orientation sensors that monitor the rotation of the boom, stick, and bucket, and track the articulation of the machine (and consequently the position of the bucket end-effector) in real-time. A generic and scalable framework and workflow for transmitting real world sensor data to update 3D equipment models inside a graphical virtual world for concurrent visualization is presented. The developed technical approach can be conceptually adapted and extended to visualize any construction operation as it occurs inside a dynamic 3D world, simply by outfitting the real equipment pieces with appropriate sensors and connecting them to their virtual counterparts.

## 2. Real-Time 3D Visualization in Digital Twins

3D visualization consists of two categories—real-time and post-processed. In the case of post-processed visualization, the input is provided entirely from a simulation model (visual simulation), or from pre-recorded data of actual resource locations (trace simulation) [16]. However, in the case of real-time visualization, particularly when an ongoing operation is concurrently represented in a virtual world (i.e., a digital twin), there is an inherent need to link the real world to the virtual world. For real-time visualization, it is essential for the position and orientation sensor data from the jobsite to update the corresponding 3D equipment models in the virtual world, in order to maintain a valid geometric display and related operational state.

The use of real-time 3D visualization and geometric proximity monitoring has key advantages over other methods, especially when considering a construction jobsite. The use of conventional video cameras for monitoring safety on the jobsite is limited by physical constraints. Video cameras can limit the field of view in relation to the jobsite and equipment [17]. Additionally, it may not be feasible to place a camera in a location that provides the best perspective view of the equipment. Real-time 3D visualization, when combined with geometric proximity monitoring, provides spatial context in addition to visual guidance, i.e., the virtual environment can help “see” obstructions (e.g., buried utilities) that might not be visible either through video cameras or by the human operators themselves. Real-time concurrent 3D visualization in digital twins is thus a key requirement for effective equipment monitoring in any context aimed toward improving jobsite safety, and rather than mere observation or recording alone.

## 3. Literature Review

Equipment tracking and monitoring can occur at the macro- and micro-level. Macro-level tracking refers to those techniques where the location of the equipment in the global space is of interest to the site or fleet manager. Examples of this are fleet tracking applications for trucks, cars, and other assets [18,19,20]. At the macro-level, the user is primarily interested in the location, but not the articulation (roll, pitch, and yaw angles) or the details of sub-component orientations. At the macro-level, it is historically common for several resources to be tracked at the same time [18,19,20].

On the other hand, micro-level tracking is defined as that which occurs predominantly at the per-equipment level, where the position of the equipment in global space along with detailed orientations of sub-components are monitored and displayed to the user for real-time visual analysis [21]. However, it must be noted that some information from the micro-level monitoring can also be used at the macro-level, such as a site manager overseeing the operations of several equipment on an earthwork jobsite by viewing the position of all equipment on site.

The authors’ proposed methodology is targeted at micro-level equipment monitoring, and the remainder of this section describes existing micro-level tracking and monitoring approaches in civil and construction engineering. The Denavit-Hartenberg notation was used by Lu and Liang [3] to develop a kinematic model for simulating the movement of a backhoe excavator, an example of articulated construction equipment. The use of ultra-wideband (UWB) technology for tracking, monitoring, and estimating the pose of cranes was demonstrated by Zhang et al. [22] by attaching UWB tags along the crane’s boom and tip. Radio Frequency (RF) technology has been implemented to provide real-time warnings to RF-tagged equipment operators and workers by triggering alarms when the distance between pieces of equipment and/or workers drops below a safety threshold [23].

The National Institute of Standards and Technology [24] developed a method to track the 3D position of a robotic crane using a laser-based 3D site measurement system to provide position and orientation information to reduce errors in an encoder-based control system, and to also map the crane’s location relative to other components in the work environment. Sensor data and related CAD models were presented to the user in a visualization system [24]. Oloufa et al. [25] have demonstrated the use of GPS technology for tracking equipment on a construction site, providing warnings to operators about impending collisions between two moving equipment. The use of real-time kinematic GPS was tested and demonstrated by Roberts et al. [26] for construction plant control and monitoring applications. Peyret et al. [27] used a combination of GPS sensors to track the motion of an asphalt paving machine’s blade by measuring its location in the vertical plane.

A combination of orientation (rotation) sensors, Global Positioning System (GPS), and laser technology has been used in applications for earthwork, grading, and compaction operations to demonstrate equipment monitoring and control [28,29]. The use of computer vision technology has been demonstrated for the recognition of construction equipment on earthmoving jobsites that has a potential to detect, track, and measure the productivity of stationary and mobile equipment [11,21,30]. The field of equipment teleoperation is an example of micro-level monitoring where the operator is not present in the equipment and controls the equipment remotely. Steffen et al. [31] demonstrated vehicle teleoperation through use of data from GPS and heading sensors by transmission over a wireless network and presentation through a 3D virtual display system.

The authors identify two primary limitations in the existing approaches. First, some of the existing methods are limited by their narrow scope in being applicable to only a specific sensor-type and/or equipment type. Being applicable to any equipment type commonly found on a construction jobsite is a key requirement due to the number of different equipment pieces that may be present on any medium to large project. Second, the monitoring of equipment without providing operators with concurrent 3D visualization and proximity monitoring information limits its effectiveness due to the limited information being presented to the operators.

## 4. Overview of the Proposed Methodology

Equipment used on construction jobsites is often articulated in nature, i.e., its individual components are linked through joints that allow rotation about their pivot. Examples of these types of equipment are hydraulic excavators, backhoe loaders, graders, dump trucks, and haulers. Monitoring such equipment involves tracking the rotation and translation that the equipment undergoes. The data collected is then transmitted to a 3D digital twin so that the real and virtual worlds can be geometrically correlated to one another. The 3D models in the virtual world, when combined with the stream of position-orientation data, can be used for carrying out proximity analysis and collision detection. Finally, the 3D visualization and related analytical output are presented to the operator. The entire process occurs in real-time or near real-time so that the output can be used by operators in their decision-making process.

**Figure 1:**
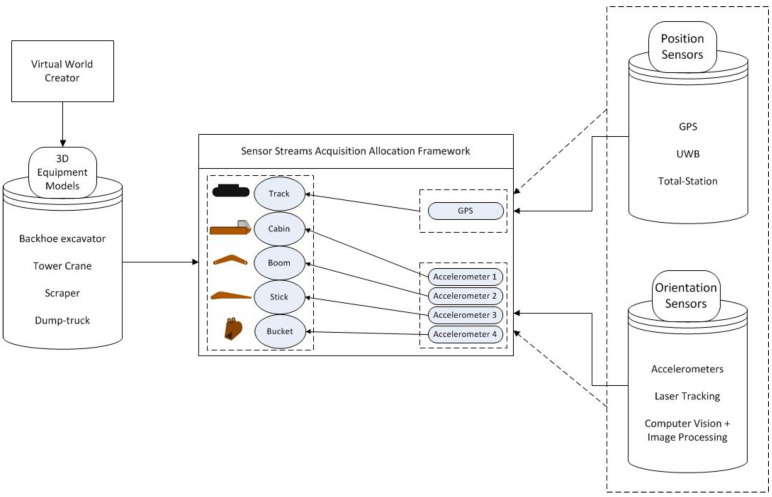
Proposed methodology and workflow for creating a link between real world sensor data and 3D virtual equipment models in digital twins.

Thus, it can be seen that being able to represent and simulate dynamic articulated equipment from the real-world jobsite concurrently in a 3D digital twin requires a link joining the real and virtual aspects of the operation [32,33,34]. There is an added level of complexity due to various types of construction equipment often being present on a jobsite, at any given instant. Similarly, an equipment’s position and its sub-components’ orientation can be recorded and monitored through a wide range of sensors and techniques. It follows that any proposed methodology must be generic in its scope if it is intended to be extensible to a broad range of projects. The proposed methodology is schematically represented in Figure 1, with the data from the real-world interfacing with individual components of the equipment in the virtual world.

Figure 1 uses an excavator model to schematically show how the equipment’s components can be connected to position and/or orientation sensor sources from the real world. A generic framework is capable of performing a similar allocation of sensor streams to different types of equipment, such as a grader, crane, or backhoe loader, based on the configuration of sensors installed on them. Similarly, the position and orientation sensors can vary from one jobsite setup to another, examples of which are presented in Figure 1. Thus, the ability to work with non-restrictive configurations of sensors and 3D equipment models is an essential part of the proposed framework and workflow.

## 5. Technical Approach

In order to be able to replicate the real-world motion of articulated equipment concurrently in the digital twin, understanding of the underlying equipment motion concepts is essential. This is primarily due to the complexity inherent in equipment such as backhoes and excavators, where it is not feasible to obtain the location of the end-effector in a direct manner [32]. These limitations in turn are caused by potential damage that the sensors can suffer if placed directly on the end-effector (bucket) where that can come in contact with earth, rocks, and soil over the course of operations. The remainder of this section describes the key technical challenges involved in concurrently replicating the translational and rotational motion of equipment inside a 3D digital twin.

### 5.1. Kinematics

Kinematics is the branch of mechanics that deals with the study of motion of a singular object or a group of objects without considering their causes [35]. The field of kinematics itself is further divided into two approaches: forward and inverse kinematics. Forward kinematics refers to the use of the kinematic equations to compute the position of an object’s end-effector from specified values for the object’s joint parameters. Inverse kinematics on the other hand is the reverse procedure of forward kinematics. It is used to compute the angles and/or lengths of individual links and joints of the object, and the rotations and translations that the object must undergo in order for the end effector to be present at a required (predetermined) final position and orientation.

**Figure 2:**
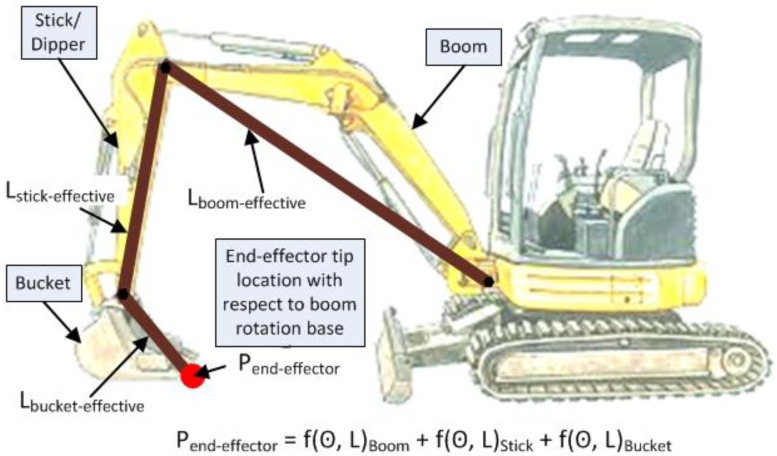
Backhoe side view with schematic kinematic chain representing its boom, stick, and bucket articulation.

In the case of equipment monitoring and operator assistance, the joint angle rotations are recorded by the sensors, and thus the end-effector’s position and orientation, P_end-effector_, is computed through a forward kinematic process where P_end-effector_ can be mathematically stated as ‘f(Θ, L)’ for every rotational joint and arm from the object’s base to the end-effector, where ‘Θ’ is the rotational angle of the joint and ‘L’ is the length of the arm rotating about the pivot for every joint. This relationship is graphically represented in Figure 2, using the example of an excavator. The reader is referred to [36] for additional discussion on the kinematic equations and process.

As seen in Figure 2, the bucket’s position and orientation depends upon the cabin’s position and the sum of the lengths and the rotational angles of the boom, stick, and bucket, respectively. The lengths of the boom, stick, and bucket are considered from pivot-to-pivot, so that the principles of forward kinematics can be applied to the computation. It must be noted that there are limitations on sensor placement upon equipment due to physical constraints and the harsh environment the equipment end-effector operates in. As a result, placing a position sensor on the end-effector that comes in contact with soil and other materials is difficult to achieve. Thus, the end-effector’s global position, i.e., position of its tip in relation to other entities on the jobsite, must be computed in an indirect manner.

It follows that determination of the global position of an end-effector has two computations associated with it. The first is the position aspect of the equipment in the environment, i.e., on the jobsite. The second involves the articulated chain of linked elements associated with the equipment. Thus, in the case of the backhoe shown in Figure 2, a GPS receiver is placed on the top of the backhoe to provide the equipment position on a jobsite in terms of latitude, longitude, and altitude. In some cases, a pair of GPS receivers are placed on the cabin of the equipment to provide a more accurate position, as well as orientation (rotation) of the cabin [26]. Data from tilt measuring sensors placed along the boom, stick, and bucket arms in combination with lengths of the respective components can provide the distance of the end-effector tip, measured from the articulated chain base, i.e., the pivot point of the boom.

Based on the placement of the position sensor on the equipment, there may be an offset between the position reported by the sensor and the base of the articulated chain. Accurate integration of the two data computations requires the inclusion of the offset distance between the base of the articulated chain and the position reported by the position sensor. The combination of every f(Θ, L) along the articulated chain provides only the position of the end-effector with respect to the base of the articulation. In order to convert this value to its global position so that the position value can be compared to other entities on the jobsite, the base pivot offset is required.

**Figure 3:**
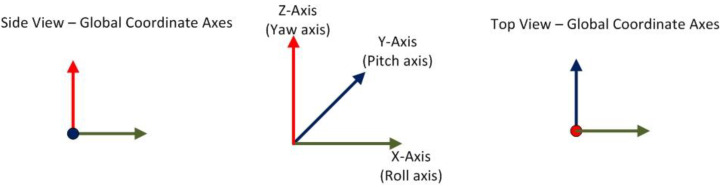
Global coordinate axes—X, Y, and Z about which a body experiences roll, pitch, and yaw respectively.

**Figure 4:**
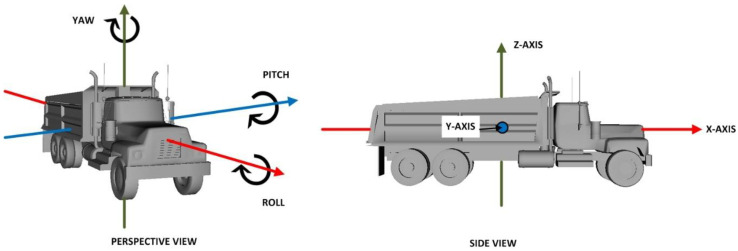
Angles described in terms of roll, pitch, and yaw about the X-, Y-, and Z-Axis in both real and virtual worlds.

Every element along the kinematic chain has a set of rotation axes associated with it. The origin of the local axes corresponds to the rotation pivot of the element under consideration. At the same time, sensors placed on the equipment components record the rotation with respect to a set of global axes that are common for all the elements comprising the equipment. Thus, there exist a set of global axes as shown in Figure 3. These axes are familiarly called the X-, Y- and Z-Axes, and are defined such that X-Axis points in the horizontal direction to the right, Y-Axis orthogonal to the plane defined by X- and Z-Axes and in a direction pointing away from the reader, while the Z-Axis points in the vertically upward direction. The three commonly used terms to describe rotation of a body in 3-dimensional space are roll, pitch, and yaw. Roll is the rotation experienced about the X-Axis, pitch is the rotation about Y-Axis, and yaw is the rotation about Z-Axis, as shown in Figure 4.

**Figure 5:**
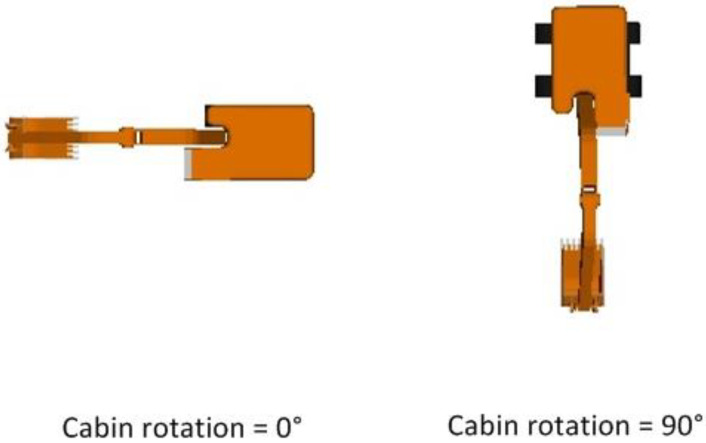
Rotation of component (cabin) results in automatic rotation of child components (boom, stick, and bucket) but not the parent component (tracks).

Equipment components in an articulated chain have a parent-child hierarchical relationship. Hence, in the case of an excavator, the track component is the parent of the cabin, boom, stick, and bucket elements. Similarly, the cabin component is the parent of the boom, stick, and bucket components. In such a parent-child relationship, any rotation or translation experienced by the parent is implicitly transferred to the child entities. For example, anticlockwise rotation of the cabin by 90 degrees about the Z-Axis, results in rotation of the cabin as well as its child elements—boom, stick, and bucket—by the same magnitude. However, the track component, which is the cabin component’s parent element, is unaffected by this rotation. This behavior is represented in Figure 5.

**Figure 6:**
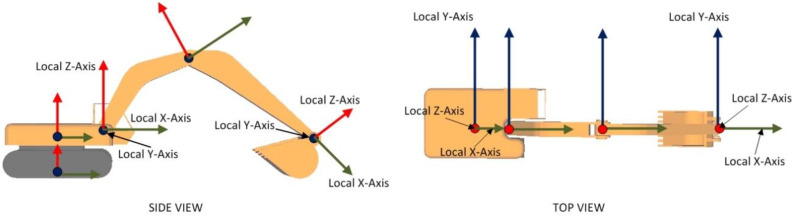
Local rotation axes for equipment components.

Due to such rotation and translation of individual components, the position and orientation of the local origin of the coordinate axes gets altered. This results in a change in direction of local X, Y, and Z axes. For example, rotation of the boom component in an anti-clockwise direction results in corresponding rotation of the stick and bucket components by the same magnitude. Due to this, the local axes direction of X, Y, and Z differ from their global directions, i.e., directions corresponding to zero translation and rotation. This is represented in Figure 6, through side and top views of an excavator.

The cumulative effect of the parent components’ rotation on a given element’s local axes represents an important parameter in computations, as the rotation angles reported by orientation sensors are typically computed with respect to absolute global axes (e.g., the horizontal and vertical directions are computed with respect to the ground surface). Thus, rotations imparted to the 3D models representing the components in the real world need to be geometrically transformed so that the real-world rotation from the sensors correlates to the existing rotation value of the 3D model component. The transformation is achieved by converting local rotations of individual components to their respective global rotation values.

### 5.2. Kinematic Equivalence

A 3D model used to symbolize equipment in the real world can represent the real world to varying levels of realism and detail. However, for 3D visualization and proximity analysis of the operation to be valid and useful to the operator, the authors have identified kinematic and dimensional equivalence as being essential requirements for a 3D model. Kinematic equivalence, in this context, refers to the property of a 3D model to concurrently mirror in the virtual world the rotational and translation motion that a piece of equipment and its sub-components undergo in the real world. This implies that the rotation or translation of a component, resulting in a position change of an end-effector in the real world, must be replicated in the virtual world in such a manner that the end-effector has the identical global position in the virtual world as it has in the real world.

**Figure 7:**
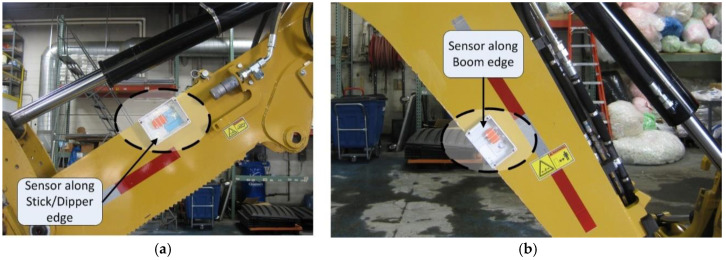
(**a**) Orientation sensors placed along the upper edge of the stick/dipper; (**b**) lower edge of the boom.

The effect of kinematic non-equivalence is most evident in the case of objects having curved bodies or objects having bends in their physical makeup, such as booms and sticks/dippers. In such objects, these physical characteristics result in differences between the axis corresponding to a certain edge and the pivot-to-pivot rotation axis. For most equipment components, the sensors are often placed along an edge of the body, as shown in Figure 7, to ensure its position remains fixed during the course of operations, and the sensors record the angle of a known edge in the real world.

**Figure 8:**
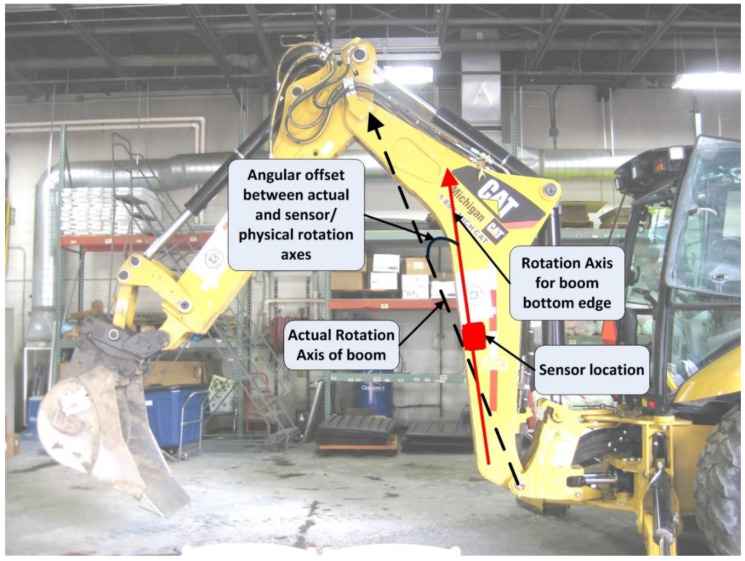
Difference in actual rotation axis and rotation axis corresponding to physical edge of object (boom).

The problems associated with kinematic equivalency are graphically represented in Figure 8. In order to ensure that the 3D model in the virtual world has kinematic equivalence to its real-world counterpart, the angular offset between the actual rotation axis (represented by dashed line in Figure 8) and rotation axis corresponding to the sensor and boom edge (represented by solid line in Figure 8) needs to be accounted for while transmitting orientation values from the real world to the virtual world. In the specific scenario of the boom object shown in Figure 8, the angular offset needs to be subtracted from the orientation sensor angle before being applied to the 3D virtual boom model. This ensures that the boom tip will have the same global position in the real and virtual worlds.

In addition to kinematic equivalence, the authors also introduce the concept of dimensional equivalence. This refers to the characteristic of a 3D model and its sub-components to represent the real-world equipment’s constituent components in size and placement. In some cases, when the 3D models used to represent equipment in the virtual world do not have identical dimensions to the real-world equipment components, the following constraints are identified as being critical. First, any sub-component forming part of the overall 3D equipment model must have identical dimensions to the real-world equipment component, such that the extents along X, Y, and Z axes (i.e., length, width, and height dimensions) are identical in the real and virtual worlds.

Through this constraint, rotation of equipment components in the virtual world results in the position of the component extremity being identical to the real world. Without dimensional equivalence, use of correct sensor rotation values will not provide the same global position for the component’s extremity, and in turn, that of the end-effector. Furthermore, for the geometric proximity analysis carried out on equipment components to provide values that are representative of the distances between the equipment and other jobsite entities (e.g., buried utilities), the dimensional extents need to be identical for the real and virtual world components.

The second constraint with respect to dimensional equivalence relates to location of base pivot joints and their height with respect to the ground surface. In the case of the kinematic chain representing the boom, stick, and bucket, shown in Figure 2, the position of the end-effector tip in global space is dependent upon the height of the joint corresponding to boom rotation pivot. Thus, in addition to accurate rotation angles and dimensions, the height of base pivot points and their distance respect to other pivot joints is critical for accurate replication of the real-world equipment operation inside a 3D digital twin.

### 5.3. Equipment Representation in a Concurrent Virtual World

As described in the previous sections, equipment monitoring is achieved through a combination of sensors placed on equipment and replication of the equipment movement (translation and/or rotation) in a 3D digital twin. This virtual world provides operators with visual assistance as well as additional spatial context information, to allow them to perform their task better. Thus, it becomes evident that representation of equipment in 3D in an accurate and real-world representative manner is a primary need for the proposed equipment monitoring approach.

Every object that is present in a 3D digital twin appears as a single cohesive entity. However, some objects are made up of one or more constituent components. Articulated construction equipment is an example of this. For instance, a construction crane consists of a cabin, boom, cable, and hook; a backhoe similarly consists of a track or wheelbase, a cabin, a front-end loader, and a boom, stick, and bucket at its opposite end. Thus, it can be seen that equipment of such type consists of more than a solitary sub-component, each of which is capable of translation, and/or rotation. The components in turn are linked to each other through a parent-child hierarchy, where translation or rotation of a parent component results in corresponding movement in a child component. This parent-child hierarchical representation is captured in a data representation structure, called scene graphs, through their layout and structure [37].

Other fields, such as computer science, commonly uses graph structures, particularly where hierarchies are required to be represented. The two types of graph data structures that exist are undirected and directed graphs. Scene graphs are one of the most widely used implementations of graphs, particularly in the field of computer graphics. Scene graphs are essentially directed acyclic graphs. They have a hierarchical tree structure for managing the object transformations, such as rotation, translation and scaling, level of detail, field-of-view culling, state transformations, and animation [38].

Scene graph implementations are typically based upon the lower-level graphics Application Programming Interfaces (APIs), such as OpenGL, and provide an additional layer of abstraction between the implementation and the underlying hardware. The authors refer the reader to [39] for more details on the use of scene graphs for creating 3D articulated equipment models. Through the use of scene graphs, equipment components can be ensured to be dimensionally and kinematically equivalent.

### 5.4. Server-Client Approach

One of the main challenges in designing effective equipment monitoring is the ability to connect a wide range of position and orientation sensors from the jobsite to 3D virtual model components for any of the commonly used equipment in the real world. Thus, scalability and non-specificity (or generality) are identified as the two key requirements for transmission and linking of real-world sensor data to the digital twin. Scalability refers to the ability to connect any number of sensors from the real world to 3D virtual equipment components in the virtual world. Non-specificity entails that sensors from the real world can transmit data and be connected to any equipment in a 3D virtual world that is representing its real-world counterpart.

The two key requirements are enabled through a server-client approach to equipment monitoring. The server refers to the part of the framework that is responsible for recording and transmitting real world sensor data in an appropriate data format to the client. The client, in this case, equates to the digital twin, i.e., 3D visualization and proximity monitoring algorithms that would consume the real-world sensor data. Insulation of the client from the sensors used on a jobsite ensures that the visualization and proximity monitoring are not restricted by the type of position or orientation sensors that may be implemented. In addition, this arrangement also allows multiple sensor data streams to be made available for the client.

The common denominators for data transfer are specified through data structures for position and orientation. The position aspect of equipment on any jobsite can be accurately described through its latitude, longitude, and altitude. This corresponds to the X, Y, and Z locations of the equipment inside the 3D digital twin. The latitude, longitude, and altitude can be specified in geographic (spherical) or projected coordinate system units in such a way that the locations of all entities are specified in the same system.

Orientation of a 3D model in a virtual world is specified through rotation about a specific axis or a combination of axes. By selection of appropriate axes of rotation in the virtual world, the rotations in real and virtual worlds can be correlated to each other. For example, the axis of rotation of sensors placed on equipment in the real world can be made equivalent to the global axes of rotation in the virtual world as described in the previous sections. Thus, the rotation values from the server are specified in terms of roll, pitch, and yaw, about the X-, Y-, and Z-Axis, respectively, as shown in Figure 4.

**Figure 9:**
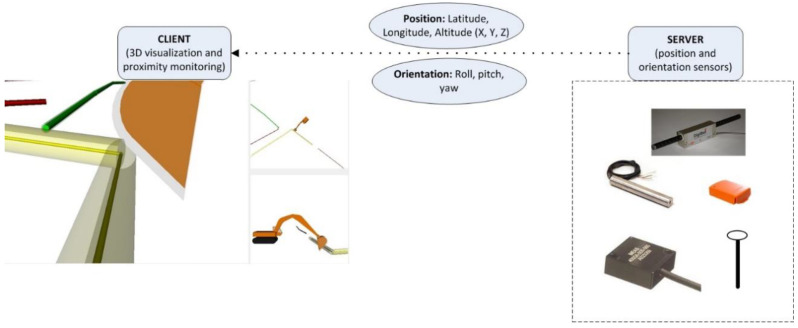
Server-Client approach for transmitting position and orientation sensor data stream from the real to virtual world.

Through the specification of orientation angles in terms of roll, pitch, and yaw, the orientation angles from the real world can always be correlated to the virtual world, regardless of the sensors used. For example, the use of linear encoders to measure the extended lengths of pistons on the server side of an equipment monitoring operation can be modified at the server-side by converting the lengths of cylinder extension to equivalent rotation angles for the boom or stick. In this manner, the same visualization and proximity monitoring-based client can be used with both direct orientation sensors, such as accelerometers, and indirect approaches, such as linear encoders. Thus, insulation of the client visualization and proximity monitoring from the sensors used on the server side ensure scalability and non-specificity, as shown in Figure 9.

## 6. Sensor Stream Acquisition Allocation (S2A2) Framework

**Figure 10:**
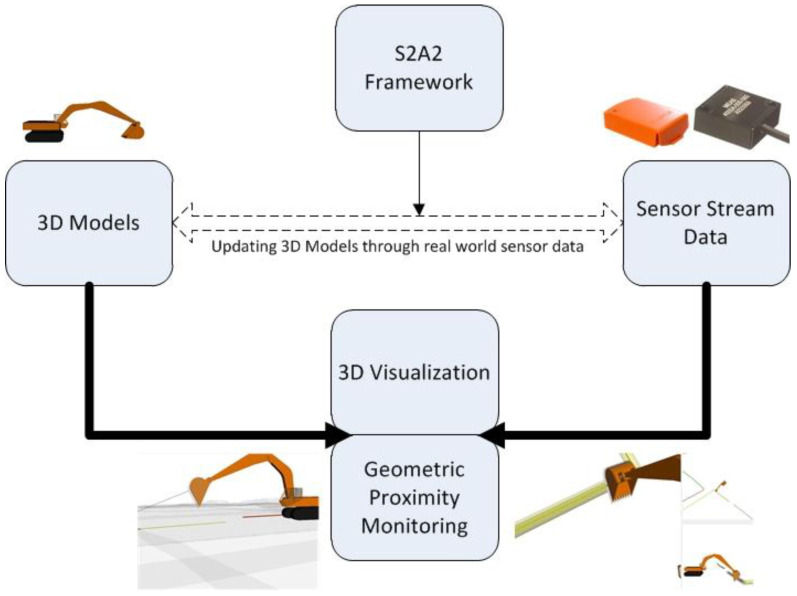
Schematic representation of S2A2 framework for integration with real-time 3D visualization and geometric proximity monitoring.

In this section, the authors introduce a computational framework developed to enable transmission of real-world sensor data into a 3D digital twin. The framework is called the Sensor Stream Acquisition Allocation (S2A2) framework. The S2A2 framework is designed as an interface to an existing real-time 3D visualization system, SeePlusPlus, that has been developed by the authors for improving safety in excavation operations through monitoring of excavators, and providing visual guidance and warnings against impending collisions with buried utilities. The S2A2 framework is a link between articulated 3D equipment models present in the virtual world (SeePlusPlus) and sensor data streams from the real world. Figure 10 shows a schematic overview of the proposed equipment monitoring approach through real-time 3D visualization and proximity monitoring.

As seen in Figure 1 and Figure 10, the process of connecting sensor data streams from the real world to 3D equipment residing in a digital twin depends upon the ability to expose to the user the available 3D equipment components that can undergo rotation and/or translation. Furthermore, the 3D equipment must exhibit the same parent-child relationship in the virtual world as is evident in the real world. 3D equipment models used for such monitoring purposes are often built from individual sub-components, rather than consisting of a single model that is incapable of movement in its sub-components. These requirements are implemented in another software tool designed for creating 3D equipment models to assist in monitoring operations. The tool is hereon referred to as the ‘Virtual Equipment Builder’ (VEB).

**Figure 11:**
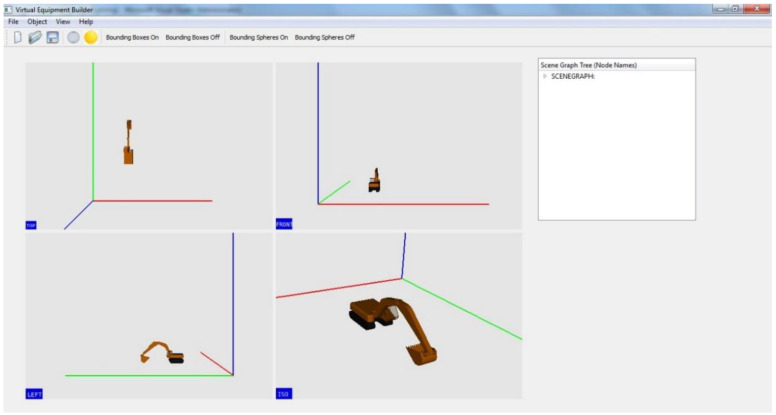
Virtual Equipment Builder screenshot showing graphical interface and multiple views of a 3D excavator being built.

VEB is designed as a graphical interface that allows users to load individual equipment components as child (or parent) elements of other components, and thus build up a complete equipment 3D model in the process. The complete equipment model can be saved for use in the SeePlusPlus real-time 3D visualization system. VEB is designed and developed as an interactive tool that allows users to manipulate the view, as well as change the position and/or orientation of components, while creating the complete equipment model. A screenshot of VEB with a created 3D excavator model is shown in Figure 11.

**Figure 12:**
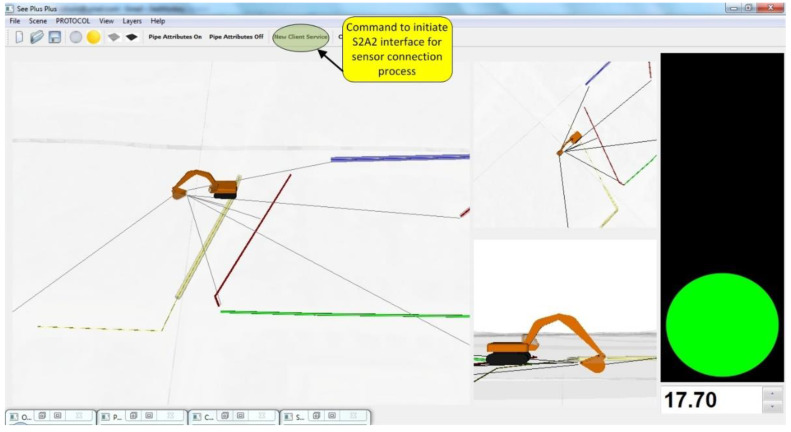
Screenshot of SeePlusPlus showing a real-time scene containing an excavator and buried utilities, associated proximity monitoring between bucket and utilities, and S2A2 initiation command highlighted.

SeePlusPlus is a 3D visualization system that provides users with visual guidance, and also has an associated proximity monitoring and collision detection module. However, for the virtual world in SeePlusPlus to be representative of real-world operations, a user requires a method to connect real world sensor data to equipment components that make up the 3D equipment models. Figure 12 shows a screenshot of SeePlusPlus displaying a concurrent scene with an excavator and buried utilities. The terrain is rendered translucent to enable visualization of otherwise occluded utilities. Proximity monitoring in the scene is displayed to the user through three channels. First, numerical shortest distance to collision; second, lines drawn between bucket and utilities; and third, traffic signal colors-based warning, showing green, amber, and red for varying levels of safety and accident risk. Figure 12 also highlights the user command to activate the sensor connection interface for making the sensor data stream available from the S2A2 framework.

**Figure 13:**
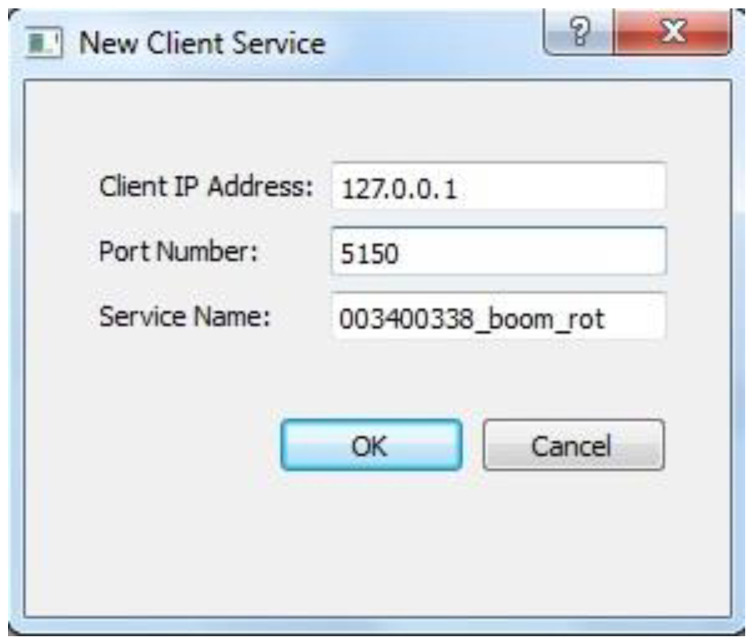
Input dialog interface for creating new client service for connecting to a server-side data stream.

The connection between server streams and the client is implemented through socket-based connections. In sensor implementations, data is transferred from the sensors to a server-side application wirelessly or through a physical connection. The server-side application then converts the raw data to position and/or orientation values that are required by the client application. Thus, the server- and client-side applications can run on the same physical machine, and the use of socket-based connections ensures that data transfer is application-independent. The command to initiate a client-side connection to the server-side application provides the user with an interface, as shown in Figure 13.

**Figure 14:**
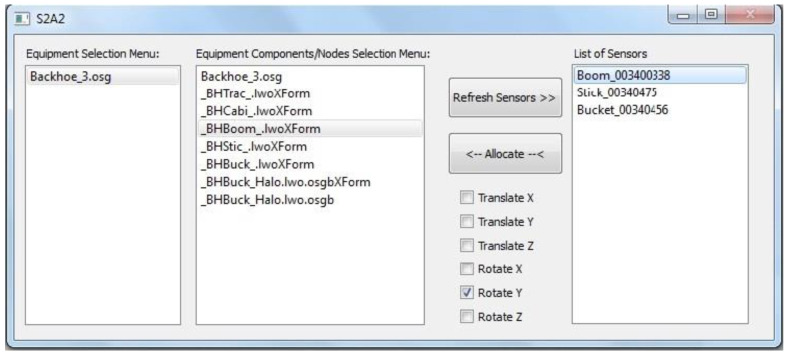
Graphical interface for user-defined connections between real world sensors and virtual equipment components.

A successful connection between client and server sides results in the new server stream being added to the list of available sensors in the S2A2 interface. The interface also presents the list of sub-components that make up the 3D equipment model. A screenshot of the S2A2 interface is shown in Figure 14. As is evident from the S2A2 acronym, the interface also allocates sensor streams to user defined equipment components. The allocation is specified through a set of checkboxes that allows users to select which component of the sensor data stream may be used to update the selected equipment component. For example, selection of only Translate X, Translate Y, and Translate Z options of a sensor stream ensures that only its position aspect would be used to update the selected equipment component.

In a similar manner, the rotation can also be specified by choosing one or more of roll (Rotate X), pitch (Rotate Y), and yaw (Rotate Z). Once an equipment component has been allocated a sensor stream, its position and/or orientation is updated in real-time, as long as the sensor in the real world is active and transmitting data. Thus, the S2A2 interface is designed to work together with the SeePlusPlus real-time 3D visualization application in providing real world sensor data to update the corresponding 3D equipment models. This is necessary to enable the monitoring of operations and improve safety.

Table 1 describes the sensors required to measure position of the excavator, as well as the orientation of its articulated components, such as boom and stick (or dipper), and rotation of the cab around the track. In the experiment, the sensors were housed in protective casings and attached using magnets to the metal components of the backhoe. In more harsh environments, a weatherproof housing would be required. In the experiment, the sensors communicated to the data collection device using a wireless Bluetooth mechanism; however, other communication techniques such as UDP can also be used.

## 7. Validation Experiments

In this section, the authors describe the procedure and setup used for carrying out validation experiments. The experiments are designed to demonstrate the functioning of the S2A2 framework and the accompanying 3D visualization when used to monitor and track a backhoe, and characterize the extent to which an arbitrary construction operation involving articulated equipment can be visualized in a real-time concurrent 3D virtual world. Through the S2A2 framework, changes in the equipment’s articulation are transmitted to a 3D visualization in real-time. This position and orientation (pose) data is then used in the visualization to update the location and pose of corresponding dynamic entities. The updated 3D equipment components are then used in real-time proximity analysis to present distance and impending collision information to the operator of the equipment.

**Figure 15:**
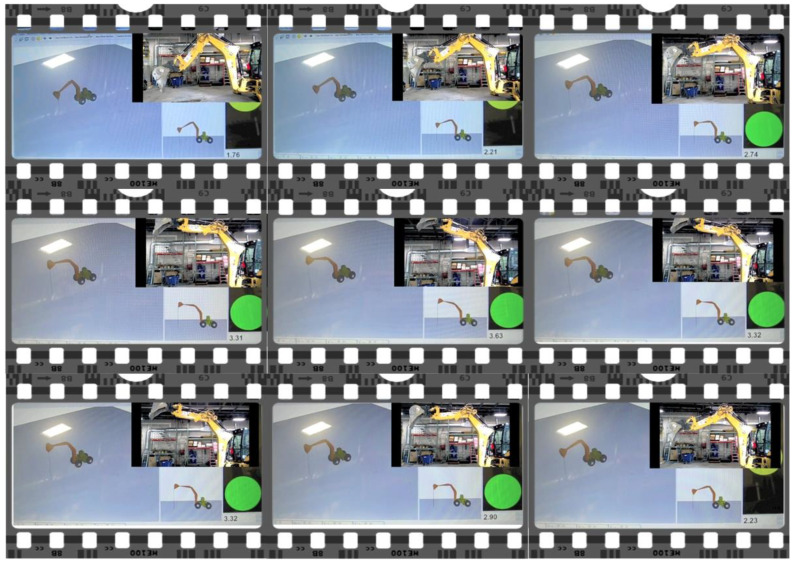
Images captured from a simultaneous video recording of the validation experiment showing backhoe in the real world and the digital twin.

The backhoe used in these experiments was a Caterpillar 430 E IT [40]. Screenshots from a simultaneous video recording of the real and virtual worlds is shown in Figure 15. The articulation of the equipment’s arm and end-effector was captured through a series of orientation sensors placed along its boom, stick, and bucket. The orientation sensors used in the experiment were XSens MTw [41]. Bluetooth wireless technology was used to transfer pose data from individual sensors to the device running the 3D visualization. The accuracy of the sensors and their calibration is measured through the proximity monitoring framework that uses the pose updates as input.

Through the 3D visualization and proximity analysis, the experiments demonstrate equipment monitoring in the following manner. In the first experiment, distances computed in the virtual world are compared to those in the real world. In the second experiment, the effect of audio and visual warnings on an operator’s performance is investigated. The rest of this section describes the experiment details and results obtained.

### 7.1. Experiment 1

The focus of this experiment was on capturing and representing the real-world equipment articulation in a 3D virtual environment. Hence, the position aspect of the equipment was discounted as the machine was assumed to be stationary. Through the proximity monitoring framework, the distance between the equipment’s end-effector (bucket in this case) and the ground surface on which the equipment was resting, was monitored at all times. If the equipment’s articulation in the 3D virtual environment was an accurate representation of the real world, the distance between the end-effector and ground surface would be identical (after accounting for sensor-based inaccuracies) in both the real and virtual worlds.

During the test, the equipment’s boom, stick, and bucket were manipulated by the operator, similarly to regular operations. As the validation required distance values from both the virtual and real worlds, the operator was instructed to stop motion of the equipment’s arm whenever a distance measurement was to be made in the real world. After the equipment had come to a complete halt, the distance between the end-effector (bucket) and the ground surface beneath it was measured using a measuring tape. This process was repeated for several different configurations of the boom, stick, and bucket. In total, 15 distance measurements were made in the real world. Corresponding distances displayed by the proximity monitoring framework in the virtual world were also simultaneously recorded. The values obtained from real and virtual world measurements are shown in Table 2.

### 7.2. Experiment 2

The second experiment was designed to test the latency of the S2A2 framework and effectiveness of the warnings provided by the 3D visualization and proximity monitoring system. The setup was similar to that in Experiment 1, where the equipment was assumed to be stationary and only orientation coordinates were used in the analysis. Unlike in Experiment 1, a tolerance check was introduced to warn the operator as soon as a preset safety threshold was breached. For the purpose of the test, distances of 2.0, 2.5 and 3.0 m were sequentially set as the safety threshold level between the end-effector and the ground surface. The proximity monitoring framework in this experiment was therefore designed to warn the operator when the end-effector came within the preset safety threshold distance to the ground surface.

Once again, the test was carried out through multiple iterations with changeable articulation of the boom, stick, and bucket. The operator was instructed to halt the motion of the equipment as soon as an audio-visual warning was provided. Once the equipment was stopped, the distance between the end-effector and the ground surface was recorded. This process was repeated ten times and results from each iteration along with the distance by which the operator had penetrated the preset buffers (2.0, 2.5, 3.0 m) are presented in Table 3. The significance of the results and their interpretation by the authors is presented in the following section.

## 8. Discussion of Results

In Experiment 1, the difference between end-effector to ground surface distance in the real and virtual worlds can be attributed to the following factors. The first and most significant factor relates to the ground surface slope. The experiment was carried out in an indoor facility owned by the University of Michigan. The floor of this facility had a slight slope/gradient to it, designed to allow water drainage. However, in the virtual world, ground surface was modeled as a perfectly flat surface due to non-availability of the indoor elevation data for the experiment site. Thus, a positive or negative slope for the ground surface in the real world would result in vertical distance values (from bucket tip to ground surface) that are less or greater than the corresponding values measured to a flat surface in the digital twin.

Second is the geometric difference between the bucket in the real and virtual worlds. The bucket of the backhoe was modeled from a pre-existing 3D model, and thus resulted in unintended dimensional variances between the bucket tip in the real and virtual world objects. The distance in Experiment 1 was measured as the vertical separation distance between the bucket teeth and ground surface. Hence, dimensional equivalence is stated as a key requirement for accurate monitoring of equipment on jobsites.

The third source of error is based on the accuracy of angles measured and reported by the orientation sensors used in the experiment. The static accuracy of the MTw sensors used in the experiment is stated as being 0.5 degrees [41]. Thus, for a boom length of 2.75 m, as in the case of the Caterpillar 430 E IT, a 0.5 degrees error results in a vertical deviation of 0.09 m. The combination of these factors is attributed to the difference between real and virtual world values for distance between the backhoe bucket and ground surface, observed in Experiment 1. The mean separation distance error with the standard deviation (shown in Table 2) is 0.06 ± 0.05 m.

Experiment 2 was designed to test the effect of audio and visual warnings on an operator when a preset safety threshold was breached by the end-effector. The experiment used safety thresholds of 2.0, 2.5, and 3.0 m. For each of the safety thresholds, the operator manipulated the equipment at varying speeds, categorized in Table 3 as ‘low’, ‘high’, and ‘very high’. Low corresponds to the angular speed when a bucket would be in close proximity to a surrounding object, or when working in a narrow or confined space. High and very high correspond to the typical angular speed of the bucket that would be observed during the course of regular excavation operations. The safety threshold value was changed throughout the course of the experiment to ensure that the operator was responding to the warnings and not the learning-effect of the same threshold value. Different depth values also provided the opportunity to analyze the effect of larger safety buffers over smaller values.

The goal of the safety buffer is to provide a warning to the operator with sufficient reaction time to bring the equipment to a halt before the end-effector can come in physical contact with an object that may be occluded, such as buried utilities covered by the soil. The effectiveness of a safety buffer is analyzed by computing the magnitude by which the buffer was breached by the bucket. Table 4 shows the list of values by which the bucket breached the preset safety threshold (expressed as a percentage of the buffer depth) after the operator was provided with warnings. In Table 4, it can be observed that the magnitude by which the safety buffer was breached did not vary significantly when the safety buffer depth was changed between 2.0, 2.5, and 3.0 m. For example, at very high operating speed, the safety buffer was breached by 67% for a 2.0 m buffer and 68% for a 3.0 m.

The average percentage safety buffer penetration for L, H, and V operating speeds was found to be 15%, 38%, and 68%, respectively. Thus, it can be seen that the safety buffer depth depends on the operating speed of the equipment and the specific operating style of a given operator. The need for a dynamic safety buffer that adjusts itself based on the operating characteristics of a given operator is identified as one of the future goals of this research. In this way, the safety buffer provided gives the operator adequate time to take evasive action once a warning is sounded. During Experiment 2, it was observed that the operator would often react late to the audio and visual warnings due to lack of previous experience with such a system. Thus, operator training with the associated monitoring systems in a simulator or actual machine is identified as an associated requirement for maximum benefit from such monitoring implementations.

## 9. Conclusions and Future Work

In this paper, the authors investigated the types of equipment monitoring that occurs on construction and mining jobsites, as well as in other commercial settings. The need for detailed or micro-level equipment monitoring was presented and the areas where its use can help reduce accidents and improve overall safety were described. The authors also presented a technical approach and workflow for equipment monitoring based on concurrent 3D visualization in digital twins, and real-time proximity monitoring using sensor-based input for updating 3D equipment components. The principles developed in the proposed methodology and technical approach were demonstrated through a prototype interface for mapping sensor data streams to specific equipment elements. This proof-of-concept interface was presented in context of a real-time 3D digital twin for assisting excavator operators in preventing unintended strikes with underground utilities.

The paper also described two validation experiments to demonstrate the ability to simulate the real-world motion of equipment in a concurrent 3D digital twin. The experiments simulate the motion of a backhoe loader’s articulated arm through orientation sensors installed on its boom, stick/dipper, and bucket. The results comparing accuracies in the real and virtual worlds are presented. In addition, the effect of a warning mechanism based on preset safety thresholds was investigated on the performance of the operator.

The authors also introduced the concept of dynamic tolerance zones based on the speed of equipment operation, which in turn depends upon an operator. It can be seen from the results in Experiment 2 that higher operating speeds require larger tolerance zones. The ability to dynamically modify the tolerance zone size as a function of operator speed is thus identified as a future direction of this research. The future goals of this research also include experimenting with tracking sensors, such as linear encoders and other sensors, that are pre-installed or retrofitted on equipment [42]. The authors are also investigating the applicability of the proposed framework on other equipment types, such as cranes, dump trucks, and graders.

## Figures and Tables

**Table 1:** List of sensors, placement on equipment, and communication techniques used/recommended.

Sensor Type	Installation Location	Quantity Measured	Communication Technique
Accelerometer	Boom	Boom rotation angle	UDP/Bluetooth
Accelerometer	Stick (dipper)	Stick rotation angle	UDP/Bluetooth
GPS	Roof of cab	Equipment position	UDP/Bluetooth
Accelerometer	Roof of cab	Cab rotation angle	UDP/Bluetooth

**Table 2:** Comparison of distance measurements made in the real and virtual world for varying configurations of boom, stick, and bucket.

Iteration No.	Distance in Real World (m)	Distance in Virtual World (m)	Real World–Virtual World (m)
1	3.07	3.12	0.05
2	1.54	1.52	−0.02
3	1.14	1.16	0.02
4	0.63	0.66	0.03
5	0.00	0.17	0.17
6	0.66	0.68	0.02
7	1.62	1.62	0.00
8	2.18	2.08	−0.10
9	2.48	2.39	−0.09
10	2.84	2.71	−0.13
11	1.82	1.87	0.05
12	0.99	0.99	0.00
13	0.61	0.67	0.06
14	2.66	2.74	0.08
15	2.54	2.59	0.05

**Table 3:** Penetration depth of safety buffer for varying operating speeds and safety buffer depths (L = Low speed, H = High Speed, V = Very High Speed).

Iteration No.	Buffer Depth (m)	Distance from the Ground Surface (m)	Penetration into Safety Buffer (m)	Operating Speed (L = Low, H = High, V = Very High)
1	2.0	1.87	0.13	L
2	2.0	1.01	0.99	H
3	2.0	0.67	1.33	V
4	2.5	2.22	0.28	L
5	2.5	1.79	1.11	H
6	2.5	0.80	1.70	V
7	2.5	1.39	1.11	H
8	2.5	1.54	0.96	H
9	2.5	1.98	0.52	L
10	2.5	2.12	0.38	L
11	2.5	1.94	0.56	L
12	2.5	2.07	0.43	L
13	2.5	1.62	0.88	H
14	3.0	2.74	0.26	L
15	3.0	2.59	0.41	H

**Table 4:** Breach of safety buffer for varying operating speeds expressed as percentage of the buffer depth (rounded to nearest whole number).

Buffer Depth (m)	Operating Speed (L = Low, H = High, V = Very High)	% Safety Buffer Breached
2.0	L	7
2.0	H	50
2.0	V	67
2.5	L	11
2.5	H	44
2.5	V	68
2.5	H	44
2.5	H	38
2.5	L	21
2.5	L	15
2.5	L	22
2.5	L	17
2.5	H	35
3.0	L	9
3.0	H	14

## Data Availability

Not applicable.
